# Morphology engineering - Osmolality and its effect on *Aspergillus niger *morphology and productivity

**DOI:** 10.1186/1475-2859-10-58

**Published:** 2011-07-29

**Authors:** Thomas Wucherpfennig, Timo Hestler, Rainer Krull

**Affiliations:** 1 Institute of Biochemical Engineering, Technische Universität Braunschweig, Gaußstraße 17, 38106 Braunschweig, Germany

**Keywords:** *Aspergillus niger*, fungal morphology, productivity, osmolality, fructofuranosidase, glucoamylase, image analysis, pellets, germination time

## Abstract

**Background:**

The filamentous fungus *Aspergillus niger *is a widely used strain in a broad range of industrial processes from food to pharmaceutical industry. One of the most intriguing and often uncontrollable characteristics of this filamentous organism is its complex morphology, ranging from dense spherical pellets to viscous mycelia depending on culture conditions. Optimal productivity correlates strongly with a specific morphological form, thus making high demands on process control.

**Results:**

In about 50 2L stirred tank cultivations the influence of osmolality on *A*. *niger *morphology and productivity was investigated. The specific productivity of fructofuranosidase producing strain *A. niger *SKAn 1015 could be increased notably from 0.5 to 9 U mg^-1 ^h^-1 ^around eighteen fold, by increasing the culture broth osmolality by addition of sodium chloride. The specific productivity of glucoamylase producing strain *A. niger *AB1.13, could be elevated using the same procedure. An optimal producing osmolality was shown to exist well over the standard osmolality at about 3.2 osmol kg^-1 ^depending on the strain. Fungal morphology of all cultivations was examined by microscope and characterized by digital image analysis. Particle shape parameters were combined to a dimensionless Morphology number, which enabled a comprehensive characterization of fungal morphology correlating closely with productivity. A novel method for determination of germination time in submerged cultivations by laser diffraction, introduced in this study, revealed a decelerated germination process with increasing osmolality.

**Conclusions:**

Through the introduction of the versatile Morphology number, this study provides the means for a desirable characterization of fungal morphology and demonstrates its relation to productivity. Furthermore, osmolality as a fairly new parameter in process engineering is introduced and found to affect fungal morphology and productivity. Osmolality might provide an auspicious and reliable approach to increase the productivity in industrial processes. Because of the predictable behavior fungal morphology showed in dependence of osmolality, a customization of morphology for process needs seems feasible.

## Background

The filamentous fungus *Aspergillus niger *is a widely used strain in a broad range of industrial processes from food to pharmaceutical industry [1-7]. One of the most prominent characteristics of this filamentous organism is its complex morphology. In submerged cultivation two distinct growth forms can be observed, the mycelial and the pelleted form [8-10]. A study of fungal morphology is highly recommended for process optimization [11]. Depending on the expressed product the optimal morphology for a given bioprocess varies [12], optimal productivity, however, correlates strongly with a specific morphological form [13-15]. This growth form not only determines the productivity of a bioprocess, but also has a significant impact on mixing and mass transfer within the bioreactor. To ensure high protein secretion and at the same time a low viscosity of the cultivation broth, it is desired by the industry to tailor-make the morphology of filamentous fungi [16]. Various process parameters and ingredients have been described in literature to influence fungal morphology [17-21], an extensive list can be found in Wucherpfennig et al. (2010) [22].

There is an abundance of literature on the effects of metal and other ions on fungal growth and metabolite production [3]. Only very few studies are devoted to the effect of so-called inert salts like sodium chloride or potassium chloride which are supposed to have no effect on metabolism. Allaway and Jennings (1970), for example, report a decrease of growth of the marine fungus *Dendryphiella salina *in the presence of 200 mM sodium and potassium chloride [23,24]. Bobowicz-Lassociska et. al. (1995) were able to increase protein secretion of washed and filtered *A. niger *mycelia by addition of KCl. An enhanced membrane permeability due osmotic pressure was argued to be responsible for the significant increase in soluble protein [25]. In addition, Fiedurek (1997) was able to increase the activity of *A. niger *expressed glucose oxidase 2.1 fold by adding 1.2 M NaCl to centrifuged mycelia, thus administering an osmotic shock to the fungus. It was further speculated that osmotic potential should be considered as a possible regulating factor in studies on the synthesis and secretion of microbial enzymes [26]. In both studies fungal mycelia were administered to an osmotic shock. No work was done on the effect of osmolality of cultivation medium on fungal growth and productivity. In the field of mammalian cell cultivation, however, an increase in osmolality is a well-known tool to increase specific productivity and sometimes final product titer [27-31]. Osmolality is generally dependent on the medium composition and changes during cultivation through accumulation of metabolic products and pH control, through addition of acid or base. Most culture media have an osmolality between 0.28 and 0.32 osmol kg^-1^.

In the present work, the influence of osmolality on morphology and productivity of two strains of *A. niger *was studied. For holistic morphologic description of fungal morphology a dimensionless Morphology number was introduced. The macroscopic morphology was successfully correlated with enzyme productivity in both strains. The expressed fructofuranosidase (EC 3.2.1.26) catalyzes the conversion of the disaccharides like sucrose into fructooligosaccharides. The market for fructooligosaccharides has great potential, due to their interesting functional properties. Their application ranges from new prebiotic products commercially available to the pharmaceutical or the diagnostic sector [32-34]. Glucoamylase (GA, EC 3.2.1.3) is a homologous protein used for hydrolysis of starch in many industrial applications [35].

## Results and discussion

### Effect of osmolality on *A. niger *productivity

**Figure 1:**
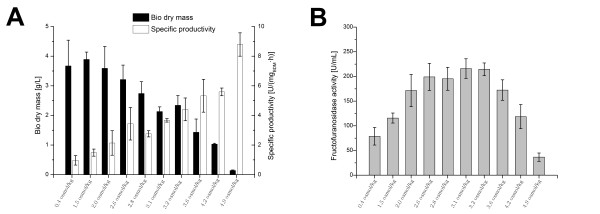
**Influence of osmolality on *Aspergillus niger *SKAn1015 productivity**. (A) Biomass dry weight (black) and specific productivity (white) after 72 hours of cultivation at 10 different culture broth osmolalities. The corresponding fructofuranosidase bulk activity [U mL^-1^] is shown in (B). Values are means for at least three 2L cultivations. Culture broth osmolality was increased permanently with sodium chloride prior to inoculation. To find the optimal osmolality of the process, more cultivations were conducted around proposed optimum at 3 osmol kg^-1^. Here, osmolality between treatments was only marginally changed. A paired t-test was conducted to test for statistical differences among treatments. Specific productivity at 3.6 osmol kg^-1 ^is not significantly different than at 4.2 osmol kg^-1^. Bio dry weight at 0.4 osmol kg^-1 ^is not significantly different than at 1.5 osmol kg^-1^. Fructofuranosidase activities at 2.6 and 2.8 osmol kg^-1^, 3.1 and 3.2 osmol kg^-1^do not differ significantly from each other.

**Figure 2:**
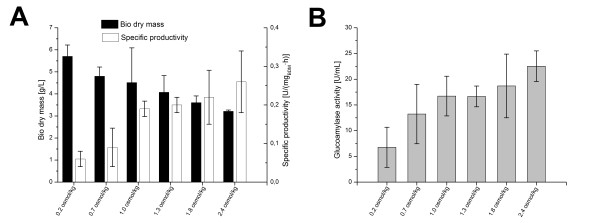
**Influence of osmolality on *Aspergillus niger *AB1.13 productivity**. (A) Biomass dry weight (black) and specific productivity (white) after 72 hours of cultivation at 6 different culture broth osmolalities. The corresponding glucoamylase bulk activity [U mL^-1^] is shown in (B). Values are means for at least two 2L cultivations. Culture broth osmolality was increased permanently with sodium chloride prior to inoculation. A paired t-test was conducted to test for statistical differences among cultivations with varying osmolality. Bio dry weight was not found to differ significantly between 0.7 and 1.0 osmol kg^-1^. Glucoamylase bulk activity at osmolality of 0.7 and 1.0 osmol kg^-1^, 1.3 and 1.8 osmol kg^-1 ^showed no significant difference.

Osmolality notably affected growth rate and conidia aggregation of *A. niger*. Fructofuranosidase and glucoamylase production in *A. niger *was, in about 50 2L cultivations, shown to be sensitive to osmolality (Figure 1 and 2). For the fructofuranosidase producing strain *A. niger *SKAn 1015, increasing osmolality within the bioreactor from the standard 0.4 to 4.9 osmol kg^-1 ^led to a considerable decline in dry cell weight from around 4 to 0.2 g L^-1^, respectively. Meanwhile the specific productivity, as defined in Methods, increased remarkably from 0.5 to 9 U mg^-1 ^h^-1 ^around eighteen fold (Figure 1A). This distinct increase is somewhat put into perspective by considering the volumetric fructofuranosidase activity in bulk as illustrated in Figure 1B. Up to an osmolality of 2.6 osmol kg^-1 ^the fructofuranosidase bulk activity is shown to increase about two and a half times. To find the optimal osmolality for the process, a considerable amount of cultivations was conducted at similar osmolality. Optimal fructofuranosidase activity of 220 U mL^-1 ^was detected at an osmolality of about 3 osmol kg^-1^. Thus osmolality between cultivations around 3 osmol kg^-1 ^was varied only marginally (Figure 1). In contrast, an osmotic pressure of more than 3.2 osmol kg^-1 ^reduces the fructofuranosidase activity per milliliter. An addition of 1.5 M sodium chloride to the cultivation medium is shown to be beneficial for fructofuranosidase production.

To check whether increasing osmolality interfered with fructofuranosidase assay, culture supernatant from a standard cultivation with 0.4 osmol kg^-1 ^was sterile filtered. In one half, the osmolality was increased up to 2.4 osmol kg^-1 ^through addition of sodium chloride, the other half was left untreated. Subsequently, the fructofuranosidase assay was performed for both samples. The samples were with 54.6 ± 0.4 U mL^-1 ^were found to have the same fructofuranosidase activity ruling out the influence of osmolality on the fructofuranosidase assay used in this study. Moreover, no significant impact on dissolved oxygen within the bioreactor could be detected (pO_2_-probe, Mettler-Toledo) while increasing the osmolality of the cultivation broth, suggesting that osmolality had no effect on the availability of dissolved oxygen for microorganisms.

Another strain of *A. niger*, the GA producing strain AB1.13 was used to test reproducibility of the beneficial effect of osmolality for enzyme production. Although both strains originate from the same clone, their cultivation performance and handling is considerably different, thus making it reasonable to compare, how both strains are affected by osmolality. Glucoamylase in *A. niger *AB1.13 is in contrast to fructofuranosidase in *A. niger *SKAn 1015 not overexpressed, thus lower activities in general were expected. The cultivation medium for this strain had an osmolality of only 0.2 osmol kg^-1^. *A. niger *AB1.13 was observed to be more sensitive towards osmotic pressure, than *A. niger *SKAn 1015. Here an osmolality of only 2.4 led to a perfectly mycelial growth. Up to 1.8 osmol/kg, however, growth of both strains was comparable. The increased osmolality led to a twofold decreased biomass and around a factor five increased specific productivity in this strain (Figure 2A). The GA activity within the bulk sample correspondingly increased about 4.5 times, while the osmolality was raised from 0.2 to 2.4 osmol kg^-1 ^(Figure 2B). *A. niger *AB1.13 was used in this study as backup to substantiate the findings made with *A. niger *SKAn 1015. Because of this and since *A. niger *SKAn 1015 is in the focus of this study a total number of only 10 cultivations with the AB1.13 strain were carried out causing a higher standard deviation in Figures 2A and Figure 2B compared with the 40 SKAn1015 cultivations. Osmolality was found to have an inhibiting effect on growth both strains, while the productivity of the biomass was considerably increased. Maximal growth rate (μ_max_) was negatively affected by osmotic pressure within the culture broth, and decreased from 0.16 h^-1 ^at standard osmolality to 0.02 h^-1 ^at 4.5 osmol kg^-1^. Bulk activity of expressed enzymes only increased up to optimal osmolality, whereas the positive effect of increased productivity outweighed the decrease in biomass.

**Figure 3:**
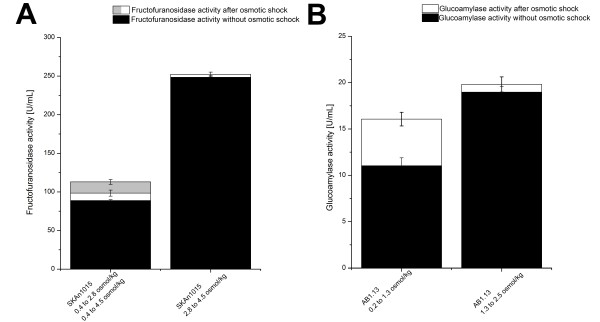
**Effect of osmotic shock on *Aspergillus niger *SKAn1015 and *Aspergillus niger *AB1.13 productivity**. An osmotic shock was administered to two *A. niger *SKAn 1015 and two *A. niger *AB1.13 samples, each with different culture broth osmolality. (A) *A. niger *SKAn 1015 was cultivated at 0.4 and 2.8 osmol kg^-1^. The 0.4 osmol kg^-1 ^sample was osmotically shocked to 2.8 and additionally to 4.5 osmol kg^-1^. The osmolality of the other sample was elevated from 2.8 to 4.5 osmol kg^-1^. (B) *A. niger *AB1.13 was grown at 0.2 and 1.3 osmol kg^-1^. The 0.2 and 1.3 osmol kg^-1 ^samples where both shocked to an osmolality of 2.5 osmol kg^-1^. All samples were incubated for one hour in shaking flasks at 37°C/30°C, 120 min^-1^. All experiments were done in triplicate. Black bars show enzyme activity after one hour incubation without osmotic shock, white and grey bars represent samples after one hour incubation in which osmolality was increased. In both cases only an osmotic shock administered to standard culture broth osmolality showed a significant elevation in enzyme activity.

According to previous works productivity could be incremented after an administration of an osmotic shock to the culture [25,26]. In this study, an osmotic shock significantly increased enzyme activity only when administered to culture broth of standard osmolality (Figure 3). In order to investigate the effect of an osmotic shock on fructofuranosidase activity, one *A. niger *SKAn 1015 cultivation at standard osmolality of 0.4 osmol kg^-1 ^and a cultivation at an elevated osmolality of 2.8 osmol kg^-1 ^were conducted. After 72 hours of cultivation biomass was collected from each cultivation. Half of the sample was subjected to an osmotic shock with sodium chloride, the other half left untreated. Both samples were subsequently incubated in shake flasks at 37°C, at 120 min^-1 ^for one hour, before measuring the fructofuranosidase activity. Experiments were conducted in triplicate. For sample cultivated initially at 0.4 osmol kg^-1^, fructofuranosidase activity was increased about 10.8%, from 88,8 to 98,4 U mL^-1^, due to an instantaneous increase in osmolality to 2.8 osmol kg^-1^. When the same sample was administered to an osmolality of 4.5 osmol kg^-1^, fructofuranosidase activity increased even further to 103.4 U mL^-1 ^(Figure 3A). No significant increase in fructofuranosidase activity could be assessed for *A. niger *SKAn 1015 sample cultivated at 2.8 osmol kg^-1 ^and shocked to 4.5 osmol kg^-1^. The GA producing strain *A. niger *AB1.13 was treated likewise. In this case, GA activity could be increased by about 50% (Figure 3B), through rapid elevation of culture broth osmolality from 0.2 to 2.5 osmol kg^-1^. An osmotic shock from initially 1.3 to 2.5 osmol kg^-1^, however, did not affect GA activity (Figure 3B). For both strains an osmotic shock was only beneficial for enzyme activity, when the fungal biomass cultivated at standard osmolality and therewith not adapted to a highly osmotic environment.

Besides sodium chloride, other inert salts were tested for increasing the osmotic pressure of the medium. Only potassium chloride yielded comparable results (data not shown). Other salts like lithium chloride, lithium bromide, caesium chloride, potassium iodide and sodium iodide repressed growth of *A. niger*.

There are several other terms for osmolality used in the literature such as osmotic pressure, water potential, water activity or salt stress. The exact term used usually depends on the method of measurement and the area of research. It is arguable, if increased productivity is due to stress physiology. Whether the term "stress" is appropriate at all, depends further on the often unknown adaptation of a fungus to a highly osmotic environment. A non-adapted species will exhibit stress physiologies, an adapted may not [36]. Many soil fungi can survive extreme external water potentials down to about -20 MPa and some *Aspergilli *are even known to survive down to -40 MPa [37]. *Aspergillus nidulans *was shown by Beever et al. (1986) to exhibit optimal growth on 0.5 M NaCl basal medium, a growth rate of 50% on medium amended with 1.6 M NaCl, and only little growth on medium amended with 3.4 M sodium chloride [38]. *Aspergilli *in general were found to be notably resistant to high salt concentrations, as they were shown to grow in the presence of 20% or more of sodium chloride [39].

The *Aspergillus *strains used in this study might therefore not necessarily exhibit stress physiologies, which is supposedly responsible for a higher rate of protein synthesis. For clarification some genetic and molecular studies should be conducted. The maintenance of intracellular pressure, also called turgor, is somewhat critical for fungi, as it is the driving force for apical expansion and therefore fungal growth [40]. Cellular osmotolerance in general depends on the maintenance of osmotic gradients across the fungal cell membrane. Cellular water potentials have to be more negative than those in the external environment to maintain inwardly directed water transport [41]. This potential is usually generated through accumulation or synthesis of osmotically active solutes by the cell [36]. This could be a reason why osmotic shocks in this study were only successful when administered to culture broth at standard osmolality. In this case, the raised extracellular osmolality is likely to have caused an external water efflux, leading to a secretion of produced enzyme. Experiments in this study support this assumption, since an increase of culture broth osmolality from 0.4 to 4.5 osmol kg^-1 ^led to a considerably higher fructofuranosidase activity, than an osmotic shock from 0.4 to 2.8 osmol kg^-1^. Fungal biomass cultivated at higher osmolality will have accumulated a sufficient amount of compatible solutes to cope with the osmotic pressure of the medium. When this adapted fungal biomass is additionally exposed to an osmotic shock, no increased enzyme activity can be measured, because the cells are already used to coping with a highly osmotic environment. However, supplementary research is needed, for further clarification in this matter.

According to literature increase of osmotic pressure has been found to alter transport of substrates [27], causing an elevation in productivity. Furthermore, increased membrane permeability and secretion is believed to be responsible for a productivity raise through increased osmolality [25,26]. In this study, only bulk activity increased with raised osmotic pressure, while biomass associated activity increased only slightly (data not shown). Hence, it is likely, that more enzyme is secreted, substantiating results of earlier studies. Results in this study suggest additionally, that a permanent increase of culture broth osmolality is more beneficial for enzyme production, than osmotic shocks, because biomass cultivated at 2.8 osmol kg^-1 ^generated much more enzyme activity than biomass cultivated in standard medium with a subsequent osmotic shock up to 2.8 osmol kg^-1 ^(Figure 3A).

Total soluble protein within the cultivation broth was considered, but not included in this study as high salinity interfered with common methods like Bradford protein assay or BCA protein assay. These problems, however, might be solved by ultrafiltration or dialysis pretreatment. In future studies soluble protein data should be included, as it provides information, whether only the produced protein or the total amount of protein is increased through high osmolality. Moreover, in future studies some protein should be purified to elucidate whether more enzyme is produced, or if the produced enzyme possesses a higher activity. A real time PCR might also be advisable, in order to clarify whether more enzyme mRNA is expressed.

### Characterization of fungal morphology

A macro morphologic approach was chosen for characterization of fungal morphology. Micro morphology was not taken into account for reasons of practicability, since an easy reproducible method to characterize fungal morphology with statistical significance was preferred. Since mycelial morphology as investigated was in clumps or pellets, parameters of particle size and shape, like projected area, perimeter, circularity, solidity and aspect ratio were applied. Circularity is a parameter to quantify the closeness to a perfect circle. It is calculated as , with a value of 1.0 indicating a perfect circle and an irregular object having a value closer to 0. Solidity is a measure of the surface of a particle, also known as roughness. It is calculated by dividing the projected area through the convex area. The convex area being the area enveloped by the convex hull perimeter, which can be illustrated as an elastic band placed around the particle, determined by the software. A smooth shape has solidity of 1.0. Irregular objects tend to have a much lower value for solidity. Solidity is a good approximation of the surface area of the fungal pellet being available for mass transport. Aspect ratio is defined as major axis divided by minor axis and is a measure of elongation of a particle. A shape symmetrical in all axes such as a circle or square will have an elongation value of 1.0 whereas elongated particles will possess considerably larger values. A complete review of sample preparation, image analysis techniques and definitions was given by Paul and Thomas in their review on characterization of mycelial morphology using image analysis [42].

**Figure 4:**
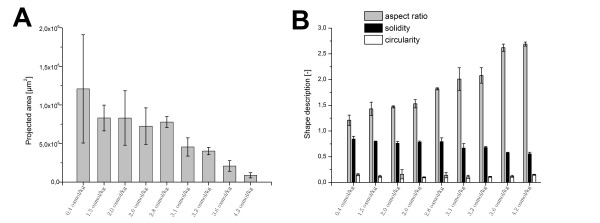
**Morphological change of *Aspergillus niger *SKAn1015 induced by osmolality**. (A) Projected area as determined by automatic image analysis is shown at 10 different culture broth osmolalities. At standard osmolality of 0.4 osmol kg^-1 ^the pellet size was generally rather heterogeneous, this led to considerably higher standard deviation. (B) Display of corresponding shape descriptors, aspect ratio (grey), solidity (black) and circularity (white) as measured by image analysis. Values are means for at least three 2L cultivations. 100 fungal particles or more were analyzed. Pictures were made after 48 hours of cultivation with a magnification 32 ×. Automatic image analysis was conducted with ImageJ 1.44 m. Beyond 4.2 osmol kg^-1 ^a perfect mycelial growth was exhibited making a macro morphologic analysis with the introduced parameters impractical.

Figure 4A reveals an influence of osmolality on pellets size. The projected area of *A. niger *SKAn1015 is shown to decline with an increase in osmolality up to 4.2 osmol kg^-1^. The standard deviation between the measured particles decreases at the same time, confirming the culture to be more homogenous at higher osmolalities. At an osmolality of 4.9 osmol kg^-1 ^a perfect mycelial morphology was found which could not be further analyzed with the introduced image analysis parameters. In Figure 4B, the influence of osmolality on parameters of particle shape is depicted. Aspect ratio and the surface parameter solidity correlate well with osmolality. The parameter circularity is not significantly influenced by the raised osmotic pressure. Fungal particles get more elongated and their surface tends to be rougher at higher osmolality. The parameter solidity describes the particle roughness, and therefore the active surface of the clump or pellet. At higher sodium chloride concentrations fungal particles have considerably more active area. At 4.2 osmol kg^-1 ^loose mycelial clumps were observed. An osmolality beyond that led to a pure mycelial morphology without any clumps or pellets.

**Figure 5:**
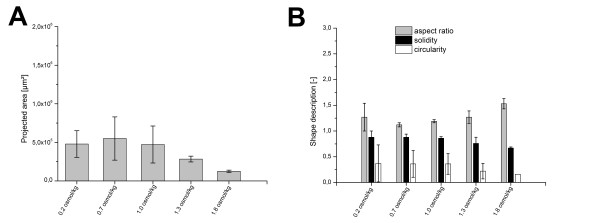
**Morphological change of *Aspergillus niger *AB1.13 induced by osmolality**. (A) Projected area as determined by automatic image analysis is shown at 5 different culture broth osmolalities. (B) Display of corresponding shape descriptors, aspect ratio (grey), solidity (black) and circularity (white) as measured by image analysis. Values are means for at least two 2L cultivations. 100 fungal particles or more were analyzed. Pictures were made after 48 hours of cultivation with a magnification 32 ×. Automatic image analysis was conducted with ImageJ 1.44 m. Beyond 1.8 osmol kg^-1 ^fungal growth was perfectly mycelial, therefore macroscopic morphologic analysis conducted up to that value only.

The response of the other *Aspergillus *strain, AB1.13 was similar, although the sensitivity of this strain's morphology towards osmolality was much greater. For this strain an osmolality of only 2.4 osmol kg^-1 ^led to a perfect mycelial morphology. As with *A. niger *SKAn1015, *A. niger *AB1.13 pellets became smaller at higher osmotic pressure (Figure 5A). Standard deviation was generally higher in this experiment, because of the fewer cultivations conducted. Figure 5B demonstrates that AB1.13 morphology was affected in the same way by osmolality as *A. niger *SKAn1015 morphology. Fungal pellets became more elongated and generally increased their surface area while osmolality was raised. Morphology of both *A. niger *strains was shown to be considerably influenced by osmotic pressure. In all cases increased osmolality led to a more mycelial growth. Such morphological response has been previously described in literature [39] and is therefore unlikely to be limited to the investigated *Aspergillus *strains. Park et al. were able to show an influence of elevated salt concentrations on *A. niger *morphology, as sodium chloride caused the swelling of the hyphal tip and branch formation within the swollen region [43,44]. In a study of Kim et al., however, potassium chloride, up to a concentration of 1 M had no effect on morphology of *Aspergillus nidulans *[45]. Other fungal strains like the halophilic *Wallemia *were found to be morphological sensitive to high salinity, as pellet size increased and wall thickness developed [46]. From the response of the examined fungi, it is rather likely that increased productivity and a more mycelial morphology are related. Image analytic methods have to be further improved; particularly in situ image acquisition as applied in Galindo et al. (2005) seems to be a promising approach [47]. Hereby the entity of fungal particles could be researched in their native state. Furthermore, a more precise structural analysis of *A. niger *morphology might be achieved by application of confocal laser scanning microscopy (CLSM) and scanning electron microscopy (SEM) providing superior surface characterization and thus allowing a more thorough image analysis [48]. Another promising approach seems to be the application of fractal analysis methods, though which the number of image analysis parameters could be possibly reduced [49].

### Correlation of morphology and productivity

Since earlier results suggested an obvious correlation between fungal morphology and protein secretion, it was an objective in this study to link productivity to morphologic appearance. The macro morphology of filamentous fungi, the process of clump or pellet formation, was already shown to have significant impact on the measured mean activities and specific productivities [50]. In addition, macro morphology determines the micro-environment of hyphae through effects on mixing, mass transfer, and culture rheology which in turn affect protein production [22]. Fungal pellets, for instance, may have dense and inactive cores due to poor diffusion of nutrients, which may lead to cell lysis and thereby loss of the interior pellet structure [51]. Microscopic morphology has other, indirect effects on productivity. Hyphal dimensions influence the secretion pathway [52] and protein secretion has been shown to be situated at the tips of fungal hyphae [53]. Wongwicharn et al. (1999) found heterologous enzyme secretion in *A. niger *to correlate with hyphal tip number as oxygen concentration was increased. Furthermore, they were able to show a correlation between the active area of the biomass and protein secretion [54]. Amanullah et al. (1999), in contrast found no direct effect of fungal morphology on protein secretion while researching the influence of agitation intensity [55].

Several parameters obtained by automatic image analysis in this study, like projected area, solidity and aspect ratio changed significantly with increased osmolality. However, too many parameters make quick assessment of fungal morphology complicated. Therefore an effort was undergone to combine relevant parameters from image analysis to a dimensionless Morphology number which can be used for a holistic characterization of morphology. Filamentous fungi can either grow as pellets or as mycelia. Between these extremes there is a whole span of intermediates, like elongated irregular pellets or clumps. Accordingly the following formula was introduced to combine the relevant morphological parameters observed:

Where A is the projected area, S is the image analysis parameter solidity, D is the maximal diameter of the pellet and E is the elongation (aspect ratio) of the particle.

**Figure 6:**
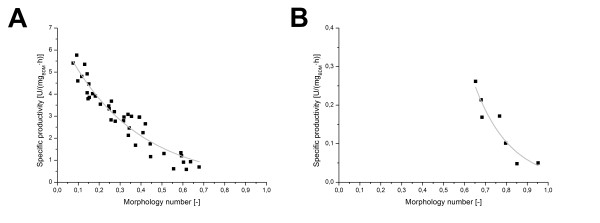
**Correlation of fungal morphology with productivity**. (A) Specific productivity of all conducted *Aspergillus niger *SKAn1015 cultivations is plotted over corresponding Morphology number (R^2 ^= 0.9). An exponential fit produced a correlation of the form *y *= 6.83 · *e*^-2.94·*x*^. (B) Specific productivity of all conducted *Aspergillus niger *AB1.13 cultivations is plotted over corresponding Morphology number (R^2 ^= 0.86). The obtained correlation is *y *= 185.69 · *e*^-5.84·*x*^. Each dot represents a single 2L cultivation.

Perfectly round and smooth pellets will in microscopic images appear as perfect circles. For such particles the Morphology number has a value of 1. The smallest fragment of mycelial morphology can be simplified as a one-dimensional line yielding a Morphology number of zero. All intermediate morphological forms will therefore have values between 0 and 1. Fairly large particles will result in a high, fungal particles with a large surface or elongated particles, in a rather low Morphology number. A fairly good correlation (R^2 ^= 0.9) is obtained when plotting the Morphology number of all *A. niger *SKAn1015 cultivations against the specific productivity (Figure 6A) with an exponential fit of the form *y *= *a *· *e*^*b·x*^. A small Morphology number therefore shows a high productivity. When the glucoamylase producing strain *A. niger *AB1.13 Morphology number is plotted against the corresponding productivity there is also a good correlation (R^2 ^= 0.86) (Figure 6B). In this case, however, notably less cultivations were conducted, as previously mentioned. Because of the more spherical particles of the AB1.13 strain, the Morphology number is slightly larger in comparison with the SKAn1015 strain.

It has to be noted, that observed changes in productivity might not be due to the change in morphology alone. The external parameter osmolality might have affected fungal physiology and through this morphology independent of each other. Observed productivity raise, however, was shown to correlate with the active surface area of the fungus, which is a good indication of plausibility. Besides osmolality several further parameters are known to influence fungal morphology, for the purpose of validation and usability the Morphology number should be tested with all of them, to proof it an authentic and reliable tool for macro morphologic description of fungal morphology.

### Influence of osmolality on *A. niger *conidia aggregation

Different morphological growth forms of *A. niger *are believed to be related to differences in spore aggregation, whereas overall growth rate has no or a quite small impact on fungal morphology. Previous experiments showed that it will be decided in the first 8 hours, depending on culture conditions, whether *A. niger *grows in pellet or mycelial morphology. Later on fungal morphology will remain constant, even if culture conditions are changed. *A. niger *at standard conditions described in this study will never develop a mycelial morphology. Mycelium grown at 4.5 osmol/kg, on the other hand, never forms fungal pellets. Even elongated fluffy pellets, which are crossover morphology, stay constant in their form and shape, if any changes in culture conditions take place after this first crucial period. Spore aggregation and subsequently germination are the most important parameters which determine fungal morphology and are therefore included in this study.

**Figure 7:**
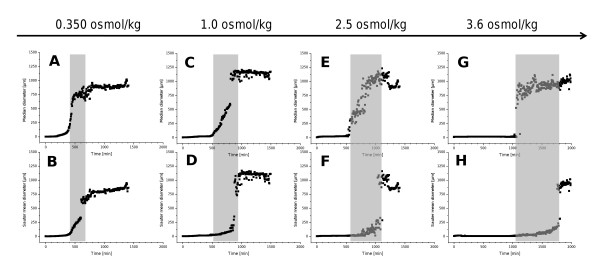
**Germination of *Aspergillus niger *SKAn1015 as effected by osmolality**. Depicted are the Median diameter (top) and the Sauter mean diameter (bottom) over time at 0.35 osmol kg^-1 ^(A, B), 1.0 osmol kg^-1 ^(C, D), 2.5 osmol kg^-1 ^(E, F) and 3.6 osmol kg^-1 ^(G, H) culture broth osmolality. The grey highlighted area marks the begin of the sudden increase of the Median diameter, till the Sauter mean diameter has reached a near constant value. The corresponding Δt-value is the germination time. Particle sizes were determined by laser diffraction.

A profound effect of osmolality on germination was revealed, while researching the aggregation of *A. niger *conidia at several osmolalities from 0.35 to 3.6 osmol kg^-1 ^(Figure 7). The median (top) and the Sauter mean diameter (SMD) (bottom) over time are given for representative cultivations. Graphics A through H show that spores germinate later at higher osmolalities. Furthermore the time difference between median and Sauter mean diameter (SMD) (grey) increases (Figure 7). Laser diffraction experiments were conducted without aeration, since bubble interfere with scattering patterns and cannot be differentiated from particles. This experiment was conducted, because conidia aggregation is believed to be one of the most important factors for development of fungal morphology. In earlier studies it was shown that oxygen requirement is very low in the first hours of germination and no oxygen limitation could be detected by measurement of dissolved oxygen [56]. Therefore omitting of aeration was feasible.

**Figure 8:**
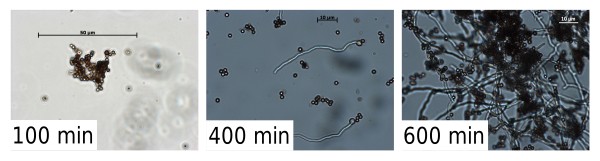
**Microscopic pictures of *Aspergillus niger *SKAn1015 germination**. Pictures with a magnification of 250 × of conidia as found in Figure 7 cultivation A/B at 0.35 osmol kg^-1^, 100, 400 and 600 minutes after inoculation.

Germination of fungal spores can be split into three steps: germination, isotropic growth (swelling) and polarized growth, due the emergence of the germ tube [57]. The cultivation broth is inoculated with spores, which are small spherical particles. After germination, small elongated particles are formed, through the formation of a singular germ tube with a tubular shape (Figure 8) [58]. Subsequently clumps and pellets are formed which are larger and again more or less spherical particles [59]. Trinci et. al (1969) first showed a pronounced lag phase and almost linear germination afterwards [60]. The same can be observed in Figure 7, graphs A to H. The definition of when a spore is germinated is based on a comparison between the length of the germ tube and the diameter of the spore. Germination time is the time until 90% of conidia have germinated, the exact percentage depending on the study [61].

In this study a novel approach was taken to examine conidia germination. Laser diffraction was employed to measure the size of sole conidia and conidia-aggregates. The method of laser diffraction generates volume equivalent distributions, which can be used to obtain statistical data, like the median. Laser diffraction, however, cannot differentiate between narrow elongated particles and larger spherical particles. Hence, germinating conidia will be recorded either as rather large particles measuring the elongated side, or spore-like size particles when measuring the considerably smaller width of the germinating conidia. The median value of a measured particle size distribution changes abruptly as soon as germination occurs and the germ tube begins to emerge. In a relatively short time frame of approximately 60 minutes the median can be observed to increase (Figure 7) from around 3 μm, the size of *A. niger *conidia, to 1,000 μm, which is the size of germinated spore aggregates and later on the diameter of pellets. The reason for the sometimes deviating median values is the measuring method of laser diffraction. Fluid dynamic properties within the measuring cell determine which side of the particle is measured, sometimes leading to considerable differences in the measured diameter. In this experimental set up the median value can be used as an indication on when the conidia start to germinate, as an emerging germ tube (Figure 8) will instantly influence the measured mean diameter. Therefore, the lag phase is the time until the abrupt increase of the median.

The Sauter mean diameter is defined as a diameter of a particle that has the same volume/surface area ratio as a particle of interest. The SMD increases later and with less velocity than the median value (Figure 7), because smaller particles are weighted more heavily than in the median value. Thus, the Sauter mean diameter in this experiment can be interpreted as an indication on when the large majority of conidia have germinated. The time difference between the median diameter and the SDM is the germination time.

**Figure 9:**
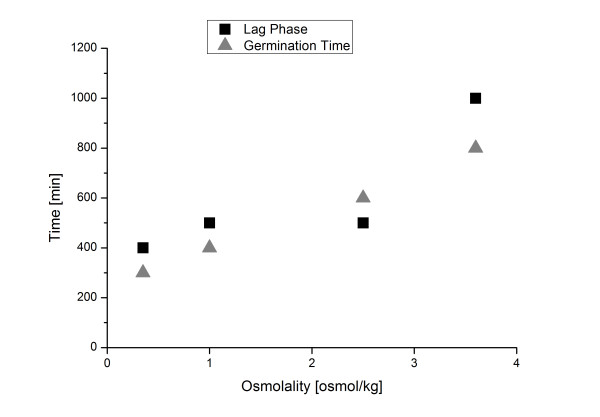
**Influence of osmolality on germination time and lag phase**. Germination time (grey triangle) and lag phase (black box) are depicted over osmolality.

In Figure 9 lag phase and germination time as effected by osmolality are displayed. Increasing the osmolality of the cultivation broth leads to a prolonged lag phase and an almost linear increase in germination time (Figure 8). Such a dependency was previously shown only on agar plates by Judet et al. (2008). The authors found that the germination time increased with decreasing water activity within the germination medium [62]. In submerse cultivations this prolonged germination process is most likely one reason for the considerable impact of osmolality on fungal morphology. Inoculum concentration was previously also shown to effect conidia germination by Grimm et al. (2004) and others. Both studies showed a prolonged germination time for increasing spore concentrations [59,63]. Inoculum concentration was also demonstrated to effect fungal morphology, as a higher number of spores led to more mycelial growth [64]. Thus at least for two parameters increased germination time leads to a more mycelial morphology. It would be interesting to determine, whether other parameters associated with mycelia morphology like low pH [65], or supplemented microparticles [17], also increase germination time of conidia. Moreover it should be an emphasis of future research, to determine, whether in submerged cultivation, a prolonged germination time and mycelia morphology are directly connected.

## Conclusions

For industrial application it is crucial to distinguish between a well and a poorly producing fungal morphology. Through the introduction of the versatile Morphology number, this study provides the means for a desirable characterization of fungal morphology and demonstrates its relation to productivity. Furthermore, osmolality as a fairly new parameter in process engineering was introduced and found to affect fungal morphology and productivity. Osmolality might provide a cheap and reliable approach to increase the productivity in industrial processes. Because of the predictable behavior fungal morphology showed in dependence of osmolality, a customization of morphology for process needs seems feasible. Since rheology of culture broth and therewith downstream processing of the product are heavily dependent on fungal morphology, a tailor-made morphology could prove to be invaluable.

A novel method for determination of germination time in submerged cultivations, by laser diffraction was introduced in this study. Thus germination time of *A. nige*r conidia was shown to increase with raised osmolality.

The Morphology number used in this study enables a very good characterization of fungal morphology. However, currently image analytic parameters in this study are adapted for characterization of inorganic particles and have their limitations when characterizing true mycelial morphology where no particles can be made out. Using the Morphology number, it is possible to distinguish between different pellet and clump morphologies in thoroughly and particle like and mycelial morphology can be differentiated. Nevertheless, it is not yet possible to compare two mycelial morphologies. To achieve this, new means have to be used. Determination of the fractal dimension also seems to be a promising method for holistic characterization of mycelial morphology.

## Methods

### Microorganism and inoculum preparation

Two recombinant strains of *Aspergillus niger *were used in this study. *A. niger *AB1.13 (van den Hondel, Universiteit Leiden, The Netherlands) is a glucoamylase producing, protease deficient, uridine auxotroph strain [66] derived from AB4.1 [67] by UV irradiation. *A. niger *SKAn1015 carrying the suc1 (fructofuranosidase) gene to produce fructofuranosidase under the control of the constitutive pkiA (pyruvate kinase) promoter was obtained from *A. niger *AB1.13 by transformation [34]. All organisms were maintained as frozen spore suspension at -80°C in 50% glycerol. To obtain spores, agar plates with the sporulation medium were plated out with spores from a frozen stock (stored in 50% glycerin at -80°C) and incubated for five days at 30°C. The sporulation medium for *A. niger *SKAn1015 contained 30 g L^-1 ^potato dextrose agar (Sigma-Aldrich) and 10 g L^-1 ^agar (Sigma-Aldrich). The sporulation medium for *A. niger *AB1.13 contained an additional 1 g L^-1 ^uridine.

After growth and sporulation, 30 mL sterile NaCl solution (0.9% w/v) was added to each agar plate, which was scraped to release the aerial mycelium. The suspension was filtered through two layers of sterile Miracloth (pore size 22 to 25 μm, Merck) to prevent agar debris and conidiophores in the spore suspension. The optical density of the spore suspension was measured photometrically at 600 nm (SmartSpec™3000, BioRad) and converted to spore concentration (m L^-1^) by a predetermined calibration curve.

### Media and cultivation conditions

A modified Vogel-Medium with the composition listed as follows (in g L^-1^) was used as the growth medium for all *A. niger *AB1.13 cultivations: glucose 20.0; uridine 0.244; 50 mL L^-1 ^salt solution containing 132 g L^-1 ^(NH_4_)_2_SO_4_; 50 g L^-1 ^KH_2_PO_4_; 4 g L^-1 ^MgSO_4_·7H_2_O; 2 g L^-1 ^CaCl_2_·2H_2_O and 0.1 mL L^-1 ^trace element solution containing: 50 g L^-1 ^C_6_H_8_O_7_·H_2_O; 50 g L^-1 ^ZnSO_4_·7H_2_O; 10 g L^-1 ^Fe(NH_4_)_2_(SO_4_)_2_·6H_2_O; 1.6 g L^-1^CuSO_4_; 0.5 g L^-1 ^H_3_BO_3_; 0.5 g L^-1 ^Na_2_MoO_4_·H_2_O; 0.37 g L^-1 ^MnSO_4_·H_2_O. The salt solution and the trace element solution were sterilized separately and added aseptically to the sterilized bioreactor with the remainder of the medium. The cultivation medium of *A. niger *SKAn1015 contained per liter 20 g D-glucose as 20 mL salt solution (6 g L^-1 ^NaNO_3_, 0.5 g L^-1 ^KCI, 1.5 g L^-1 ^KH_2_PO_4_, 0.5 g/L MgSO_4_·7H_2_O) and 1 mL trace element solution (10 mg/L EDTA, 4.4 mg L^-1 ^ZnSO_4_·7H_2_O, 1.01 mg/L MnCl_2_·4H_2_O, 0.32 mg/L CuSO_4_·5H_2_O, 1 mg/L FeSO_4_·7H_2_O, 0.32 mg L^-1 ^CoCl_2_·6H_2_O, 1.47 mg L^-1 ^CaCl_2_·2H_2_O, and 0.22 mg L^-1 ^(NH_4_)6Mo_7_O_24_·4H_2_O). Carbon source, salt, and trace element solutions were autoclaved separately at 121°C for 20 min and chilled to room temperature prior to mixing and use.

Batch cultivations were carried out in a 3-L stirred tank bioreactor (Applikon, Schiedam, The Netherlands) with two six-bladed disc turbine impellers. Bioreactors were inoculated with a suspension of freshly harvested conidia to give a spore concentration of 1·10^6 ^mL^-1 ^after inoculation. All bioreactor cultivations were carried out at least in triplicate. Growth temperature was 37 ± 0.1°C (*A. niger *SKAn1015) and 30 ± 0.1°C (*A. niger *AB1.13). Aeration rate (1.0 L min^-1^), agitation speed (200 min^-1^) and pH value (pH 5.0) were automatically kept constant.

### Biomass concentration, osmolality, enzymatic assays and statistical analysis

The biomass dry weight was measured gravimetrically by filtering (Nalgene 300-4000) a defined amount of biomass suspension through a pre-dried (48 h at 105°C) and pre-weighted suction filter (Filter Discs Grade 389, Sartorius). Prior to drying (48 h at 105°C), the filter was rinsed several times with deionized water to remove medium components from the biomass. The biomass dry weight concentration (g L^-1^) was calculated as the difference between the weight of the filter with and without dried biomass divided by the sample volume.

In this work the unit osmolality (osmol kg^-1^) instead of the more common osmolarity (osmol L^-1^) was used, since osmolality, lacking a volumetric unit, is not as temperature sensitive as osmolarity. The osmolality of the standard medium was 0.35 osmol kg^-1^. To examine the effect of osmotic pressure this standard osmolality was adjusted permanently to the desired value by addition of sodium chloride. Osmolality was monitored regularly during cultivation. For determination of osmolality, 50 μl of bulk sample were analyzed by freezing-point depression using an Osmometer (Osmomant 030, Gonotec).

Glucoamylase (GA, 1,4-α-D-glucan glucohydrolase, EC 3.2.1.3) activity was analyzed with the PNPG (4-nitrophenyl-α-D-glycopyranoside, Fluka) method described by Withers et al. [68] based on a spectrophotometeric analysis of 4-nitrophenol, which is formed by the reaction of GA with the colorless PNPG and appears yellow under alkaline conditions with an absorbance maximum at 400 nm. Extraparticulate GA activity was determined by measuring the enzyme activity in the culture supernatant obtained by sterile filtration (minisart syringe filter, Sartorius). Intraparticulate activity was quantified by separating the pellets of a given sample volume from the culture supernatant, washing them with deionized water and disintegrating them with a mortar mill (RM100, Retsch) in the equivalent buffer volume. The suspension obtained was assayed for its enzyme activity.

The specific activity of fructofuranosidase of bulk culture was quantified by filtration of 1.5 mL culture broth through a cellulose acetate filter (pore size 0,2 μm, Sartorius). The reaction mixture (220 μL) consisted of 200 μL 1.65 M sucrose dissolved in 0.05 M phosphate buffer (pH 5.4) and 20 μL sample. Reaction was initiated by addition of 200 μL sucrose to a 20 μL sample and incubated at 40°C for 20 min. The reaction was stopped by heating at 95°C for 10 min. After cooling, the reaction mixture was centrifuged at 13,000 g for 10 min at 4°C. Glucose formed from cleavage of sucrose by fructofuranosidase was then quantified using a GOD/POD assay (Sigma-Aldrich) [69], which was modified for high-throughput analysis in 96-well microtiter plates (MaxiSorp, Nunc, Langenselbold, Germany). For this assay an enzyme reagent solution was prepared by re-suspending 10.5 mg glucose oxidase (GOD) and 3 mg of peroxidase (POD) in 90 mL of 0.05 M phosphate buffer (pH 7.0) and 10 mL of 95% ethanol containing 25 mg of o-dianosidine, respectively. Subsequently, the reagent solution was filtered through a cellulose acetate filter (pore size 20 mm, Sartorius) and immediately used for the assay. For this purpose, 2 μL sample was mixed with 200 μL of the reagent solution in a microtiter plate well. After 10 min incubation at room temperature, glucose was quantified indirectly through absorption measurement at 450 nm using a 96-well Sunrise microplate reader (TECAN, Crailsheim, Germany) and the XFlour4 data retrieval software. To account for residual glucose in the culture broth, negative controls were carried out by using samples in which fructofuranosidase was inactivated by heating at 95°C for 10 min prior to incubation. All enzymatic assays were done in triplicate.

To test for statistically significant differences between cultivation of different osmolality the Monte Carlo method was applied [70]. Sample data, within the standard error previously determined by experimentation, was evaluated using Matlab (MathWorks, United States). The data sets were used in a paired t-test conducted with Origin (Originlab, United States), to test for statistical difference between data sets.

### Microscopy and image analysis

Throughout the cultivation culture morphology was monitored offline using a stereo-microscope (Stemi 2000-C, ZEISS, Jena, Germany) with an AxioCamMRc5 camera (ZEISS, Jena, Germany). The pellets were suspended individually in a Petri dish filled with diluted cultivation medium, several images of fungal particles were acquired. Around 100 pellets were analyzed for each sample. Image analysis and characterization was carried out using the ImageJ 1.44 software that is available in the public domain [71]. ImageJ was chosen in this study for its robustness and versatility and the more than 400 plugins available to fit the program to personal needs [72]. Each image was converted into an 8-bit grey-scale image. Subsequently a grey-level threshold, calculated by ImageJ using the Otsu algorithm, was applied, resulting in a binary image which was later analyzed using the inbuilt analyze particles function. Only macromorphologic parameters were considered for image analysis. The morphological parameters evaluated were projected area, perimeter, circularity, solidity, and aspect ratio for mycelial aggregates (clumps and pellets).

### Parameters used for determination of cultivation performance

Cultivation performance was in general judged by the activity of the produced enzyme. As units, the yield per volume of cultivation broth (U mL^-1^) and specific yield per biomass cell weight U mg^-1 ^were measured at the end of cultivation. For determination of specific productivity the growth curve of the microorganism, using biomass dry weight (BDW), as biomass was integrated, yielding the biomass dry weight integral (BDWI).

The BDWI was therefore calculated by:

Subsequently the specific productivity (U mg^-1 ^h^-1^) was obtained by plotting the enzymatic activity of the enzyme over the BDWI, providing an approximate linear relationship with the slope being equal to the specific productivity of the process:

### Measurement of particle size distribution for characterization of spore aggregation

A laser diffraction technique (Mastersizer 2000, Malvern) was applied to analyze the aggregation of spores. This system basically comprised a helium-neon laser (λ = 632.8 nm) as a radiation source, which illuminated the dispersed pellets in the measuring zone and a series of detectors to measure the light pattern produced over a wide range of angles. The lower limit of the obscuration was set to 5%, which was high enough to achieve a good signal to the noise ratio; the upper limit being 30%, which was below the limit defined by the detector dynamics and the algorithm treating multiple scattering. The cultivation medium (dispersant) was assessed to possess the optical properties of water. Both scattering angle and scattering intensity were dependent on the pellet size. The diffraction of the laser light on the dispersed pellets resulted in a specific diffraction pattern, which was collected by the detectors and recorded by the software (Mastersizer 2000, version 5.60). By applying an appropriate optical model (Fraunhofer Approximation) to calculate the scattering pattern and a mathematical de-convolution procedure, the volumetric particle size distribution that best matched the measured scattering pattern was calculated.

For measurement of particle size, culture broth was pumped permanently from a 2-L stirred tank stirred tank bioreactor with a double Rushton turbine (Applikon, Schiedam, Netherlands), maintained at pH 5, without aeration, through the Mastersizer measuring cell. A peristaltic pump specifically designed for gentle pumping (Pro-280 MCP, Ismatec) was used in the process. In early experiments the pump was shown to have no influence on particle size. Sample measurement time was 15 seconds, resulting in a total of 15,000 snaps to be averaged, with an interval of 285 seconds between measurements. Around 400 measurements were made during a cultivation, which was conducted until pellet formation was completed at a median particle diameter of around 1,000 μm.

## Abbreviations

BDW: biomass dry weight; BDWI: biomass dry weight integral; D_max_: maximal pellet diameter; GA: glucoamylase; SMD: Sauter mean diameter; μ_max: _maximal growth rate

## Competing interests

The authors declare that they have no competing interests.

## Authors' contributions

TW had the idea for the study, did the literature review and prepared the manuscript. He also did some of the analysis, supervised the experiments and did the aggregation experiments. TH did the main lab work, cultivations and enzyme essays. Figures were prepared by TW and TH together. RK supervised the study, and participated in its design and coordination and helped to draft the manuscript. All authors read and approved the final manuscript.
